# Immunogenic Properties of *Streptococcus agalactiae* FbsA Fragments

**DOI:** 10.1371/journal.pone.0075266

**Published:** 2013-09-24

**Authors:** Salvatore Papasergi, Veronica Lanza Cariccio, Giampiero Pietrocola, Maria Domina, Deborah D’Aliberti, Maria Grazia Trunfio, Giacomo Signorino, Samuele Peppoloni, Carmelo Biondo, Giuseppe Mancuso, Angelina Midiri, Simonetta Rindi, Giuseppe Teti, Pietro Speziale, Franco Felici, Concetta Beninati

**Affiliations:** 1 Metchnikoff Laboratory, University of Messina, Messina, Italy; 2 Department of Molecular Medicine, Unit of Biochemistry, University of Pavia, Pavia, Italy; 3 Department of Diagnostic, Clinical and Public Health Medicine, University of Modena and Reggio Emilia, Modena, Italy; 4 DiBT, Department of Biosciences and Territory, University of Molise, Pesche, Isernia, Italia; University of Padova, Medical School, Italy

## Abstract

Several species of Gram-positive bacteria can avidly bind soluble and surface-associated fibrinogen (Fng), a property that is considered important in the pathogenesis of human infections. To gain insights into the mechanism by which group B Streptococcus (GBS), a frequent neonatal pathogen, interacts with Fng, we have screened two phage displayed genomic GBS libraries. All of the Fng-binding phage clones contained inserts encoding fragments of FbsA, a protein displaying multiple repeats. Since the functional role of this protein is only partially understood, representative fragments were recombinantly expressed and analyzed for Fng binding affinity and ability to induce immune protection against GBS infection. Maternal immunization with 6pGST, a fragment containing five repeats, significantly protected mouse pups against lethal GBS challenge and these protective effects could be recapitulated by administration of anti-6pGST serum from adult animals. Notably, a monoclonal antibody that was capable of neutralizing Fng binding by 6pGST, but not a non-neutralizing antibody, could significantly protect pups against lethal GBS challenge. These data suggest that FbsA-Fng interaction promotes GBS pathogenesis and that blocking such interaction is a viable strategy to prevent or treat GBS infections.

## Introduction

The Gram positive bacterium *Streptococcus agalactiae* (group B Streptococcus, GBS) is a frequent colonizer of the intestinal and genital tracts of humans and a leading neonatal pathogen [1,2]. Maternal colonization with GBS is the primary risk factor for life-threatening neonatal infections, including pneumonia, sepsis and meningitis. Moreover, GBS frequently causes arthritis, endocarditis and sepsis in adults with underlying chronic disease and in elderly people [3]. The pathogenic potential of these bacteria is dependent on the expression of a large variety of surface-exposed virulence factors [4]. Colonization and invasion of host barriers is, at least partially, related to the ability of GBS to bind human fibrinogen (Fng) [5,6,7] and strains causing severe invasive infections can strongly interact with this protein [8]. Fng is present at high concentrations in plasma and in the extracellular matrix and binds to host cells via a number of signaling and non-signaling receptors [9]. Therefore, Fng can act as a molecular nexus between pathogens and human tissues and can modulate a number of host cell functions, particularly those involved in inflammatory responses and coagulation [10].

The ability to bind Fng has been classically linked, in GBS, to the expression of two surface proteins, FbsA and FbsB, with their relative importance varying in strains belonging to different clone types [11,12,13]. More recently, it was found that the Srr1 glycoprotein also contributes to Fng binding [14]. It is possible that FbsA is sufficient for binding to epithelial and endothelial cells, but not for cell invasion, a process for which FbsB [15] or Srr1 [14] are also required. Moreover, FbsA mediates platelet aggregation, which likely plays a role in GBS-induced endocarditis [16]. Despite the potential importance of FbsA in the pathogenesis of GBS disease, the mechanisms by which this factor binds Fng and contributes to virulence are poorly understood. FbsA displays a variable number of tandem repeats and a wall-anchoring region. Deletion of *fbsA* resulted in decreased virulence in a murine model of septic arthritis [17]. However, neither active immunization with the N-terminal portion of FbsA nor passive immunization with a neutralizing anti-FbsA antibody had protective effects in that model [17], suggesting a minor role, if any, of Fng binding in the virulence properties of FbsA. In contrast, in a recent study, passive immunization with polyclonal or monoclonal antibodies protected mice against systemic GBS challenge [18]. Therefore it is presently unclear whether FbsA can be a target for immunization strategies to prevent GBS infection.

We describe here the isolation and functional properties of FbsA protein fragments identified by screening genomic GBS phage displayed libraries for the presence of Fng binding clones. We found that maternal immunization with one of these fragments conferred protection to offspring against lethal challenge with GBS in a mouse model that closely mimics human neonatal disease. Notably, immune protection in this model was mediated by anti-FbsA antibodies and could be recapitulated by administration of a monoclonal antibody that was capable of neutralizing Fng binding, but not by a non-neutralizing antibody. Our data suggest that blockade of FbsA-mediated Fng binding may be a viable strategy in controlling GBS disease and that FbsA fragments may be useful in the development of a GBS vaccine.

## Results

### Selection of GBS display libraries

**Figure 1:**
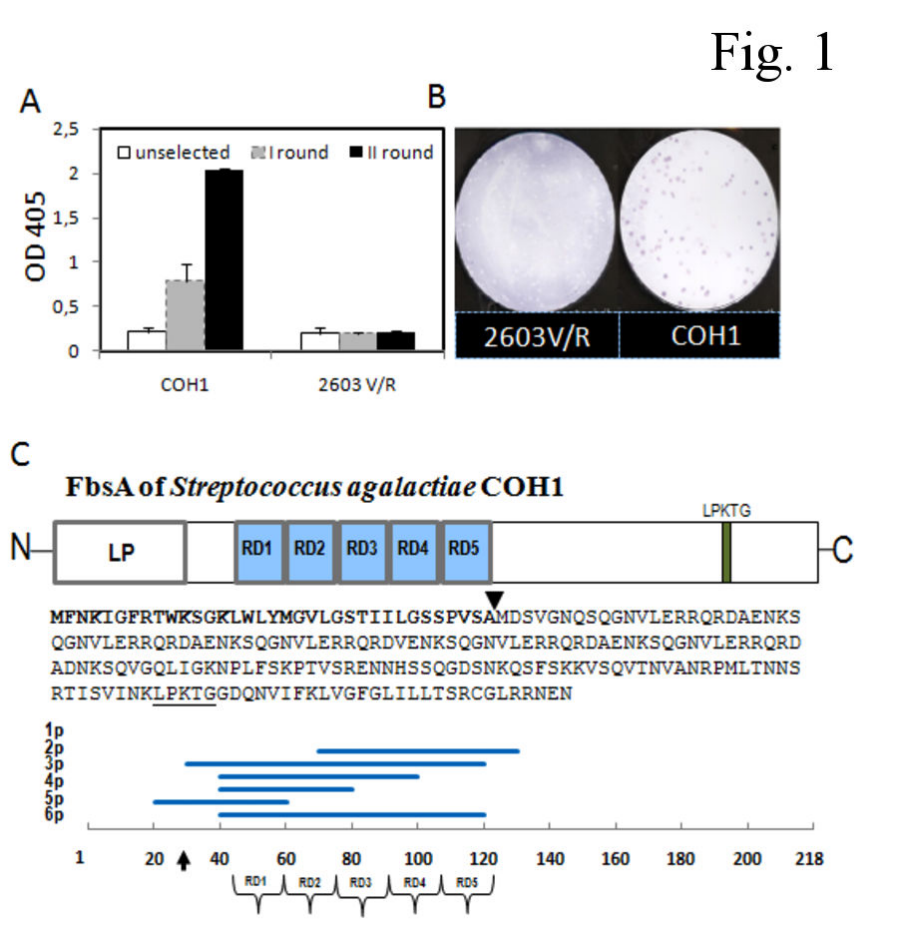
Selection and screening for Fng binding of phage displayed genomic GBS libraries obtained from the COH1 and the 2603 V/R strains. **A**. Reactivity of GBS libraries obtained after the indicated selection rounds. After each selection round using Fng-coated beads, libraries were tested for Fng binding using a phage ELISA assay (see Materials and Methods). Columns and error bars represent means + standard deviations of three replicate determinations. **B**. Plaque immunoscreening of Fng-selected COH1 and 2603 V/R libraries showing individual Fng-binding clones as colored dots. **C**. Schematic representation of FbsA fragments from Fng-binding clones. Top to bottom: organization of the FbsA protein encoded by the *fbsA* gene in the genome of GBS strain COH1; predicted FbsA amino acid sequence; position of the fragments (1p-6p) encoded by phage inserts along the FbsA sequence. The horizontal axis represents amino-acid position. Arrows indicate the predicted leader peptide (LP) cleavage site. N-, N-terminal end; RD1-RD5, repeat domains 1-5; LPKTG, cell wall anchoring motif; C-, C-terminal end.

Two different phage display libraries were constructed using partially digested genomic DNA from *S. agalactiae* strains COH1 and 2603 V/R (serotypes III and V, respectively) and affinity selected using Fng-coated magnetic beads. In a phage ELISA assay, an increasing Fng binding of phage pools after each selection round using the COH1, but not the 2603 V/R, library was observed (Figure 1A). Consistent with this, no Fng-binding clones were detected by plaque screening of the 2603 V/R library after selection, while 38 clones were isolated from the COH1 library (Figure 1B). After these positive clones were assayed by PCR, six different insert size groups were identified and representative clones from each group (designated as 1p, 2p, 3p, 4p, 5p, and 6p) were selected for sequence analysis. All of these sequences matched *fbsA* in the COH1 genome and were predicted to encode protein fragments of the N-terminal region of FbsA (Figure 1C). The complete sequences of these inserts are reported in Table S1. Each of the fragments contained one to five repeat units of an amino acid motif, GNVLERRQRDA(V) E(D) NKSQ, implicated in Fng binding [11]. In the 2603 V/R genome the *fbsA* locus (*sag1052*) encodes a truncated protein that lacks the Fng-binding repeats, perhaps explaining our inability to isolate Fng binding clones from the 2603 V/R phage display library.

### Fng binding activity of FbsA fragments

**Figure 2:**
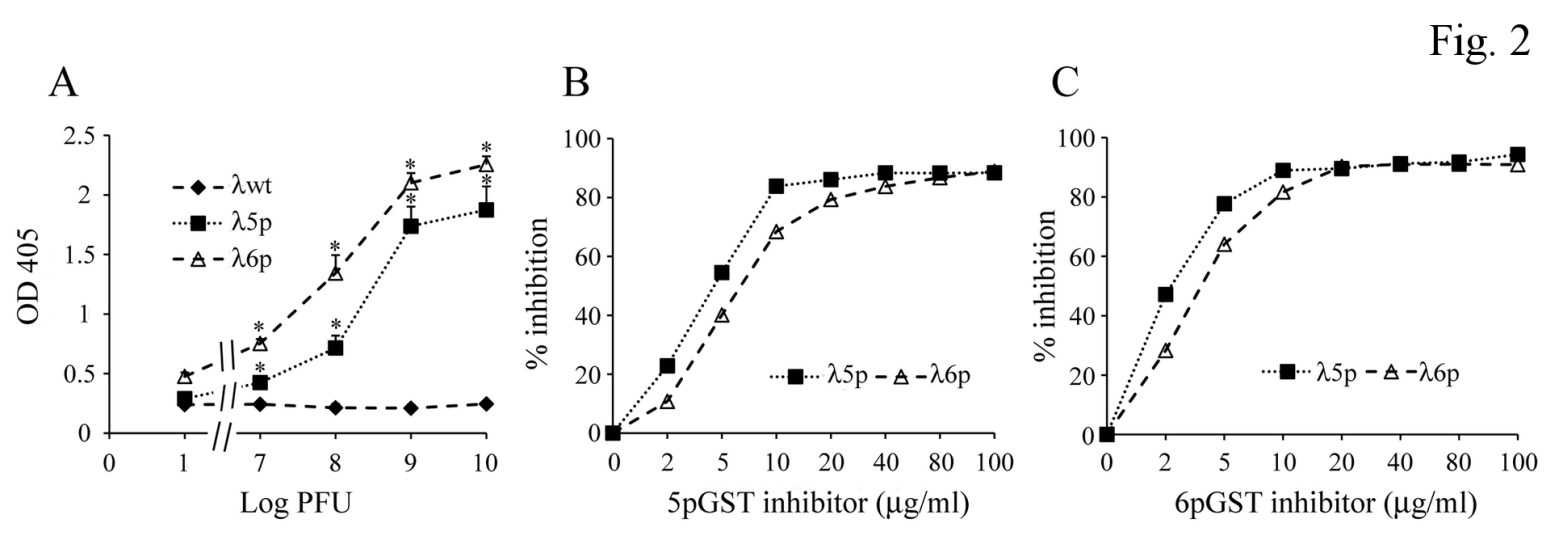
Binding of Fng to phage particles in the presence and in the absence of inhibitors. **A**. Binding to Fng of increasing numbers of 5p or 6p lambda phage (λ5p or λ6p) particles. Plates were coated with Fng, and phage particles were added at the indicated PFU numbers followed by anti-lambda phage rabbit IgG and alkaline phosphatase-labeled goat anti-rabbit IgG. Error bars represent means ± standard deviations from three independent experiments; *, p<0.05 by analysis of variance followed by the Student Newman Keuls test. **B** and **C**. Inhibition of binding of 5p or 6p lambda phage particles (λ5p or λ6p, 10^8^ PFU) to immobilized Fng in the presence of increasing concentrations of recombinant FbsA fragments (5pGST and 6pGST in panels B and C, respectively) used as inhibitors. Data are from one experiment, representative of three producing similar results.

**Figure 3:**
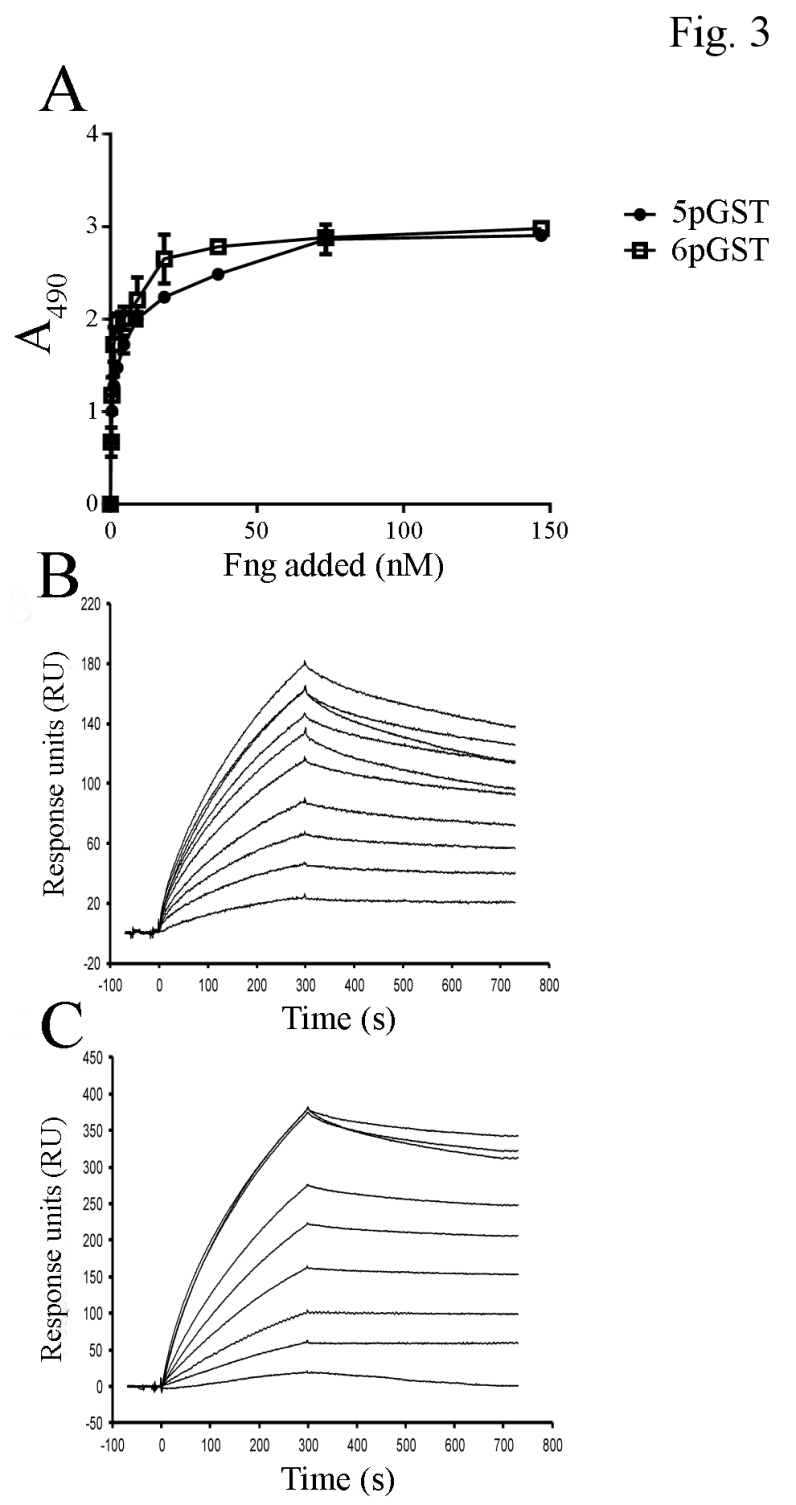
Interaction of Fng with recombinant FbsA fragments. **A**. Dose-dependent binding of Fng to recombinant FbsA fragments. 5pGST and 6pGST were coated onto microtiter plates (500 ng/well) and incubated with increasing amounts of Fng, followed by mouse anti-Fng IgG and HRP-conjugated rabbit anti-mouse IgG. Values represent the means of triplicate samples ± S.E. This experiment was performed three times with similar results. **B** and **C**. Surface Plasmon Resonance analysis of the interaction of 5pGST and 6pGST with Fng. 5pGST (panel B) and 6pGST (panel C) were captured on a BIAcore sensor chip coated with goat anti-GST IgG. Human Fng (2.92 nM to 750 nM) was then flowed over the chip surface. The data shown are representative of three individual experiments.

To analyze the ability of FbsA fragments to bind Fng, we selected two phage clones, 5p and 6p, whose inserts encode one and five repeats, respectively. As reported in Figure 2A, when increasing particles from each clone were added to surface-coated Fng, a saturable binding was observed, as detected using anti-phage antibodies. This prompted us to express 5p and 6p as recombinant proteins in fusion with GST. In a competitive binding assay, both 5pGST and 6pGST proteins efficiently inhibited binding of phages to immobilized Fng (Figure 2, panels B and C). The direct binding of 5pGST and 6pGST to Fng was also tested in an ELISA format. As shown in Figure 3A, Fng bound to both surface-coated 5pGST and 6pGST in a saturable manner, with half maximal binding values of 31.2 ± 3 and 63 ± 3 nM, respectively. It was previously established that the presence of an increasing numbers of repeats in FbsA results in enhanced Fng binding [11]. However, it is presently unclear whether this effect is related to increased binding affinity or merely to the ability of multiple repeats to bind multiple Fng molecules. To discriminate between these possibilities, the binding affinities of 5pGST and 6pGST to Fng were determined by SPR. To this end, 5pGST or 6pGST were immobilized on a sensor chip, over which different concentrations of Fng were subsequently flowed. The results from equilibrium analysis revealed K_D_ values of Fng for 5pGST and 6pGST of 27.5 ± 4.7 and 23 ± 2.4 nM, respectively (Figure 3, panels B and C). These data demonstrate that the one-repeat 5pGST and the five-repeat 6pGST FbsA fragments bound Fng with similar affinities, suggesting that the presence of multiple repeats does not result in increased affinity of FbsA-Fng interaction.

### Active immunoprotection

**Figure 4:**
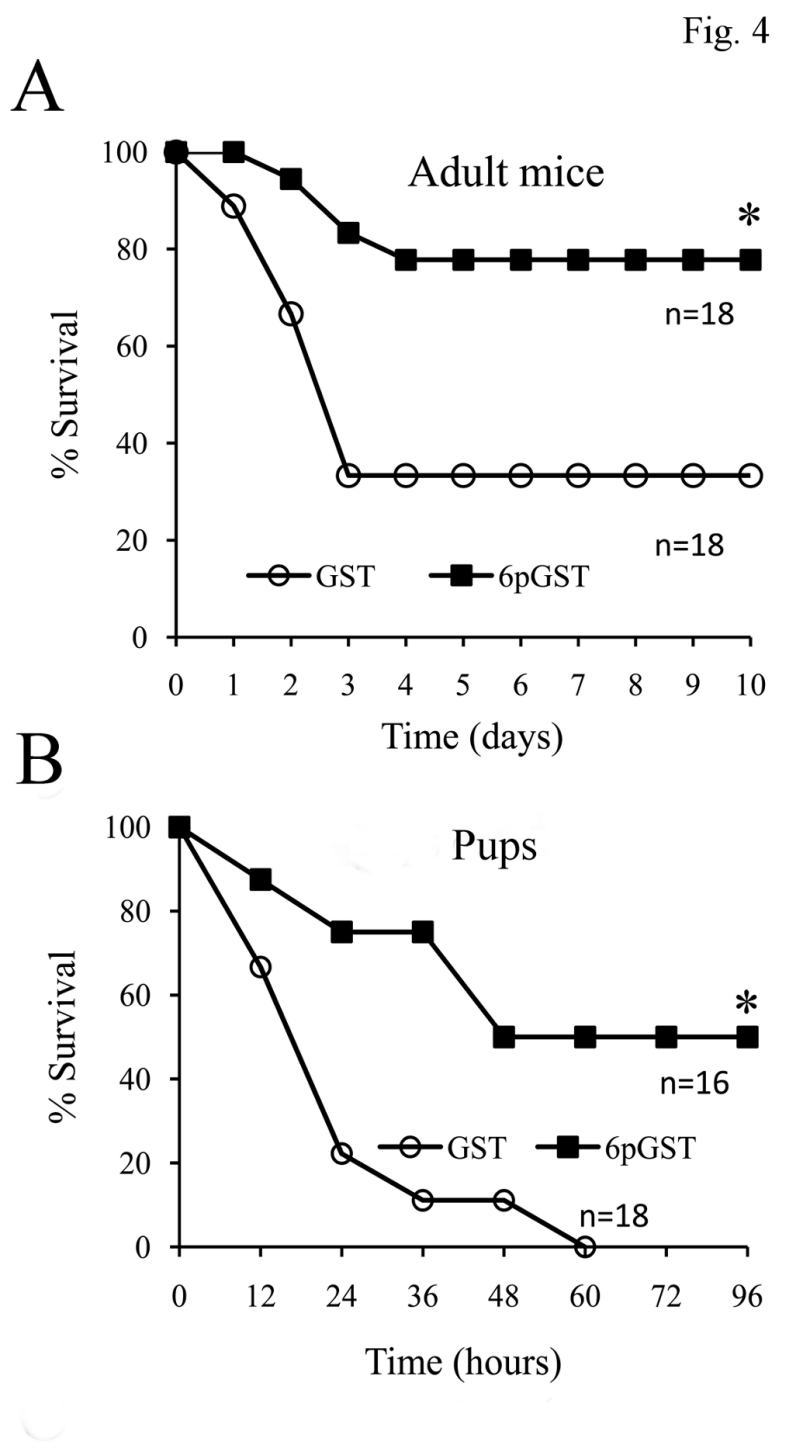
Effects of active immunoprotection with the 6pGST FbsA fragment in adult and neonatal mouse models of GBS sepsis. **A**. Immunoprotection in adult mice. Five-week-old CD1 mice underwent three immunizations with the 6p FbsA fragment fused to GST (6pGST) or with GST alone. At 3 weeks after the last immunizations mice were challenged by the i.p. injection of GBS strain COH1 (5x10^7^ CFUs) and lethality was observed daily. *, p<0.05 relative to GST-immunized mice by Kaplan-Meier survival plots. Shown are the cumulative results of two independent experiments. **B**. Effect of maternal immunization on survival of experimentally infected pups. Female CD1 mice (5 wk old) were immunized three times with the 6p FbsA fragment fused to GST (6pGST) or with GST alone. Mice were then time-mated and two-day-old pups were infected s.c. with 250 CFUs of GBS strain 6313. *, p<0.05 relative to GST-immunized mice by Kaplan-Meier survival plots.

So far, the effects of active immunization with FbsA have been tested in one study only [17]. Thus, it was of interest to explore the immunoprotective activity of our FbsA fragments in different GBS sepsis models. To this end, the five-repeat fragment (6pGST) was used to immunize adult mice and, after three administrations, all mice had 6p-specific serum antibody titers ranging from 1:8,000 to 1:64,000 (data not shown). Mice were then challenged i.p. with 5 x 10^7^ CFU of the COH1 strain at 3 weeks after the last immunization and lethality was observed for 14 days. Under these conditions, immunization with 6pGST resulted in 77% (14 mice out of 18) survival, while only 33% (6 mice out of 18) of the GST-immunized animals survived (p< 0.01) (Figure 4A). In addition, blood colony counts were significantly lower in 6pGST-immunized mice at 18 h after challenge (Figure S1). Due to the inconsistence of our data with those of the previous study cited above [17], we next investigated whether 6pGST immunization could afford protection against infection caused by the same GBS strain (i.e. the 6313 strain) used in that study. Moreover, since the goal of anti-GBS vaccination is to induce placentally transferable antibodies, we ascertained whether maternal 6pGST immunization could protect mouse pups against GBS challenge in a stringent model that closely mimics naturally occurring neonatal infection [19]. Female mice were immunized with 6pGST or GST as detailed above and time-mated. Their offspring were then challenged with the 6313 strain at two days of age. In a first experiment, 15 of 19 (79%) neonatal pups born to dams vaccinated with 6pGST survived challenge with 100 CFU, compared with 43% of those born to GST-immunized control mothers (p<0.05, data not shown). In a second experiment (Figure 4B), 50% of neonatal pups born to dams vaccinated with 6pGST survived GBS challenge, while all of those born to GST-immunized control mothers died (p<0.05). In each experiment, survival rates between litters within a test group were similar (data not shown).

### Passive immunoprotection

**Figure 5:**
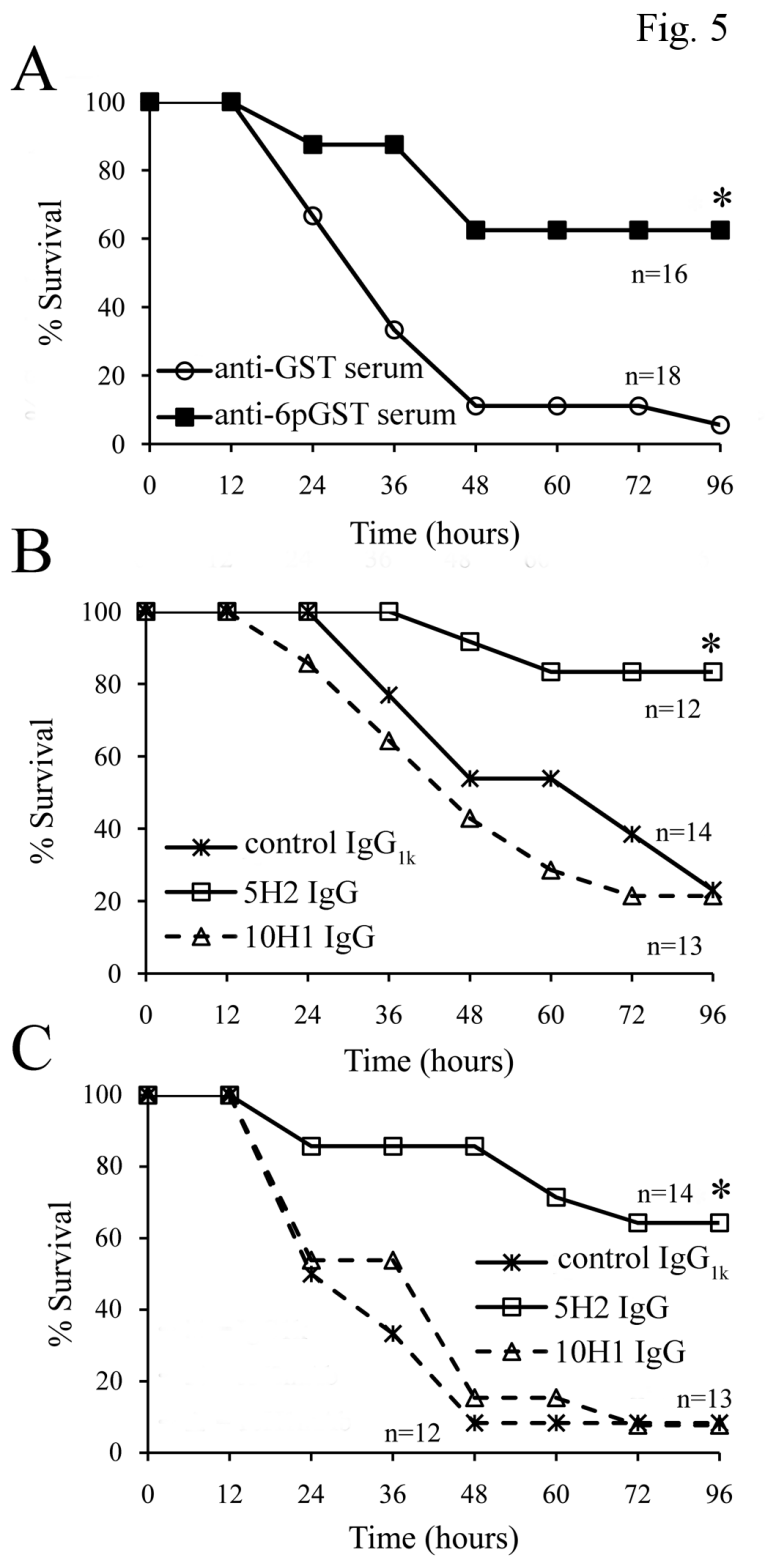
Effects of passive immunization in GBS sepsis models. **A**. Effect of administration of sera from 6pGST- or GST-immunized adult animals. Two-day-old pups born to unimmunized mothers were administered with immune sera (diluted 1:10) via a s.c. route. After 3 h, pups were infected s.c. with 250 CFUs of GBS strain 6313. *, p<0.05 relative to anti-GST serum treated-mice by Kaplan-Meier survival plots. **B** and **C**. Effects of passive immunization with anti-FbsA mAbs 5H2 and 10H1 (both IgG_1k_). Two-day-old pups born to unimmunized mothers were administered 50 (B) or 10 (C) µg of mAb 5H2, mAb 10H1 or mouse IgG1 (isotype control) via an s.c. route. After 3 h, pups were infected s.c. with 250 CFUs of GBS strain 6313. *, p<0.05 relative to IgG_1k_- or 10H1mAb-treated mice by Kaplan-Meier survival plots. Each panel summarizes the results of one independent experiment.

To clarify the mechanisms by which maternal 6pGST immunization protected offspring from GBS infection, we first ascertained whether protection could be induced by the exogenous administration of immune sera. Serum samples from 6pGST- or GST-immunized adult mice were pooled and used to passively immunize two-day old pups. Each litter was divided in 2 groups consisting of pups given either anti-6pGST or anti-GST serum. In a first experiment, all pups receiving anti-6pGST serum survived a challenge dose (100 CFU of the 6313 strain) that killed 56% of the anti-GST-treated animals (p<0.05, data not shown). In an additional experiment, 62% and 1% of, respectively, anti-6pGST and anti-GST-treated pups survived infection (Figure 5A, p<0.05). The above-described protective activities of anti-6pGST serum could be related to a variety of antibody-dependent functions, including bacterial opsono-phagocytic killing, neutralization of FbsA-mediated Fng binding or both. To gain further insights on this, we took advantage of availability of a panel of mAbs raised against a synthetic analog of the FbsA repeat motif ( [16], G.P. and P.S., unpublished results). From this panel, we selected two IgG_1k_ mAbs, 5H2 and 10H1. Of these, 5H2, but not 10H1, was capable of completely preventing binding of soluble Fng to 6pGST (Figure S2), as well as GBS adherence to immobilized Fng (Figure S3). The selected mAbs were next used to prevent lethality in the neonatal model of GBS disease. As reported in Figure 5, panels B and C, both 10H1 and the control IgG1k did not affect of the pups. In contrast, the neutralizing 5H2 mAb markedly protected neonates against GBS-induced lethality using challenge doses that killed nearly all of the control pups. These results indicated that the protective activities conferred by anti-FbsA antibodies were likely related to their ability to neutralize Fng binding. To further exclude the possible role of 5H2 in the phagocytic process, 5H2 F(ab’)_2_ fragments were prepared and tested for protection in a passive immunization experiment. As shown in Figure S4, administration of equimolar amounts of whole 5H2 IgG or 5H2 F(ab’)_2_ fragments determined a similar pattern of survival of GBS-inoculated pups. Overall, these data indicated that the protective effects of mAb 5H2 against GBS infection were not due to bacterial opsonization or other Fc-dependent functions, but rather to neutralization of FbsA-Fng interactions.

### Effects of *fbsA* deletion on GBS virulence

**Figure 6:**
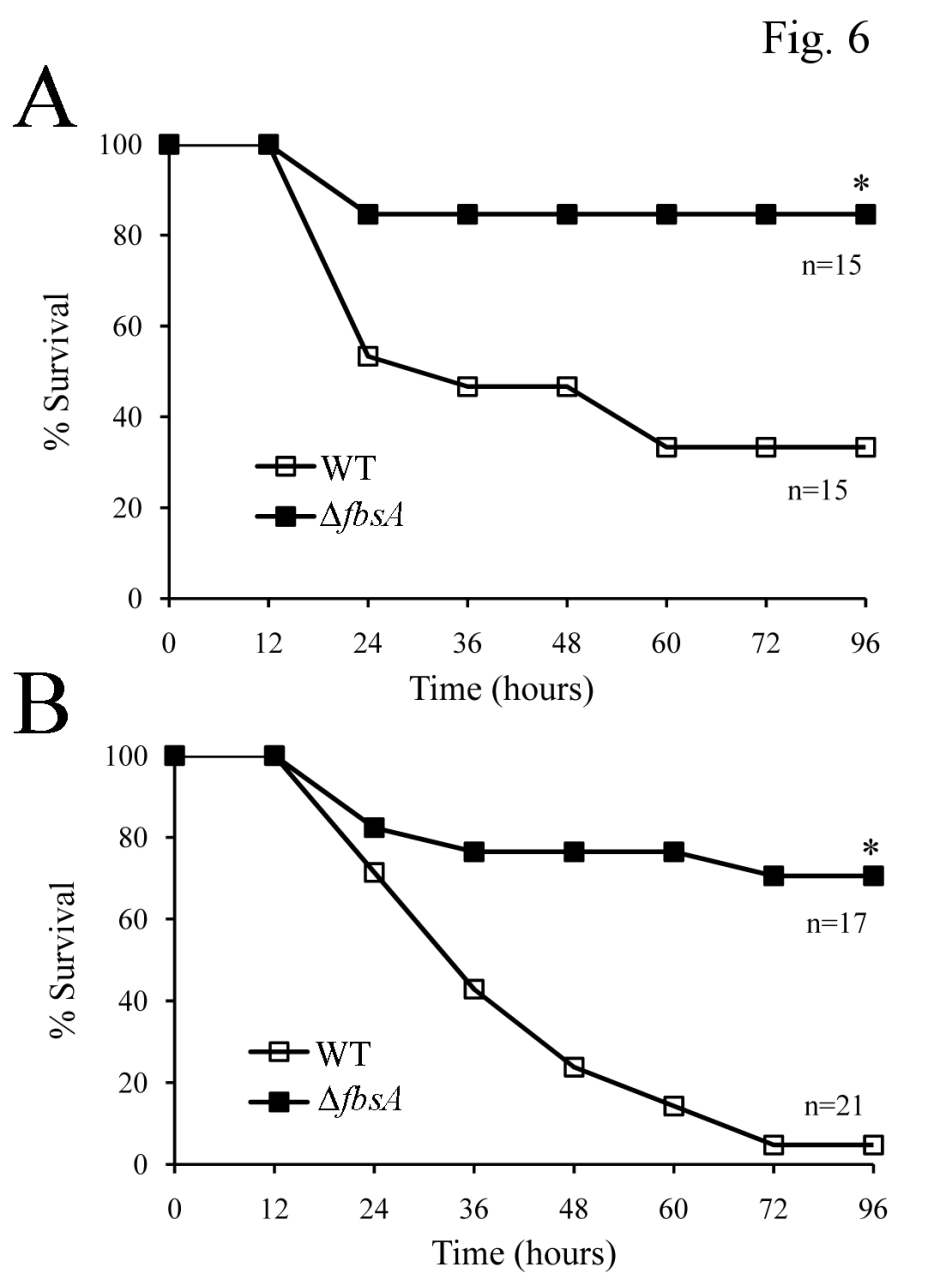
Effects of deletion of the *fbsa* gene on GBS virulence in adult and neonatal mouse models of GBS sepsis. A. Eight-week-old CD1 mice of either sex were injected i.p. with 2x10^8^ CFUs of GBS strain 6313 or of its *fbsa*-deleted (Δ-*fbsa*) mutant. Shown are cumulative survival data from two independent experiments. *, p<0.05 relative to wild-type by Kaplan-Meier survival plots. **B**. Two-day-old CD1 pups were infected s.c. with 250 CFUs of GBS strain 6313 or of its *fbsA*-deleted (Δ-*fbsA*) mutant. *, p<0.05 relative to wild-type by Kaplan-Meier survival plots. Shown are cumulative survival data from two independent experiments.

The impact of *fbsA* deletion on GBS virulence has been previously studied only in a septic arthritis model in adult mice [17]. Therefore we next investigated whether FbsA deficiency affects the outcome of GBS-induced lethal sepsis and whether such effects differ in neonatal, as compared to adult, disease models. The latter point was of particular interest, since neonatal hypersusceptibility to GBS may be linked to a relative complement deficiency [20] and Fng bound to the surface of group A streptococci can inhibit complement deposition [21]. Neonatal and adult mice were infected with a previously described GBS mutant bearing a deletion in the *fbsA* gene [11,16] and with the parental wild type 6313 strain. As expected, much higher doses of the wild type strain were required to induce lethal infection in adults, compared to two-day-old pups (2x10^8^ and 250 CFU, respectively, Figure 6). However, a similar loss of virulence of the Δ*fbsA* strain, relative to the parental 6313 strain, was observed in the neonatal and in the adult models (Figure 6). These data indicated that FbsA has an important role in the pathogenesis of GBS sepsis and that the extent to which virulence is affected by the absence of this protein is similar in adult and neonatal mice.

## Discussion

The intrapartum administration of antibiotics to colonized women has been associated with decreased incidence of GBS infections during the first week of life in several countries [22]. Despite this, GBS persists as a major public health issue worldwide and as a frequent cause of disease in neonates, in adults with predisposing conditions and in the elderly [20]. Since it can potentially prevent GBS-induced disease in all age groups, including stillbirths, vaccination represents the most effective and sustainable long-term preventive strategy. Clinical trials have indicated that immunization with capsular polysaccharides conjugated with tetanus toxoid is effective in inducing anti-capsular antibodies capable of enhancing phagocyte-mediated bacterial killing [20]. Moreover, much attention has been recently devoted to the identification of protein antigens of GBS that may be useful, either alone or as carriers in polysaccharide-protein conjugates, to increase the efficacy and the strain coverage of anti-GBS vaccines [23,24]. The main finding of the present study is that a Fng-binding fragment of the GBS protein FbsA has immunoprotective activity, which is likely mediated by the induction of neutralizing anti-FbsA antibodies.

In several bacterial pathogens, the capability to bind host Fng has been associated with an increased ability to cause invasive disease [7]. After screening genomic GBS libraries for Fng binding, we isolated several sequences of DNA encoding FbsA fragments. In contrast, sequences of other GBS proteins that are also known to bind Fng, such as FbsB [12] or Srr1 [14], were not detected despite the fact that the corresponding genes were present in the genomes used to construct the libraries. The reasons for this are unclear, but may be related to a number of hypothetical factors including a bias against the expression of certain proteins by our phage libraries or a relative lack of sensitivity of our immunoscreening assay, which may preferentially detect high-affinity interactions. It is interesting to note, in this respect, that the affinity for our FbsA fragments of Fng, as measured in the present study, is considerably higher than that reported for whole FbsB [12].

In the present study, we focused on the immunoprotective activities of an FbsA fragment, designated 6p. Notably, active maternal immunization with 6p significantly protected pups from lethal GBS challenge and such protection could be recapitulated by the administration of sera from 6p-immunized adult animals to pups born to unimmunized dams. These data were not apparently in accordance with a previous study where active immunization with the N-terminal portion of FbsA, or passive immunization with an anti-FbsA mAb, did not ameliorate the outcome of septic arthritis in mice. It is likely that the remarkable difference between the present study and the previous one is related to the diverse experimental models used. For example, in our models, GBS was injected. i.p. or s.c. and replicated in the inoculation site to subsequently spread into the blood while in the work performed by Jonsson et al. bacteria were directly injected into the bloodstream and colonization of distant organs such as the joints and the kidneys was measured. It is possible that anti-FbsA immunization is more effective in controlling local replication and subsequent systemic spreading, than in preventing hematogenous colonization of target organs.

Moreover, different disease manifestations were used to evaluate the outcome of infection in the two studies. Here, we looked at irreversible signs of septic shock and death, which always occurred within 4 days after inoculation, while in the Jonsson et al. study outcome was evaluated on the basis of weight loss and clinical and histological signs of arthritis [17]. Therefore, anti-FbsA immunization may be more effective in controlling rapidly evolving, life-threatening infections such as those observed in human neonates, as compared to more slowly evolving hematogenous arthritis, typically observed in adults. Finally, it should be noted that different immunogens (an FbsA fragment encompassing only the Fng-binding region *vs* the whole N-terminal FbsA portion) were used by the two groups. There are several documented instances in which inoculation with a relatively small fragment of a larger immunogen, but not the larger immunogen itself, resulted in protective anti-bacterial immunity [25,26]. Future studies will be needed to verify whether this phenomenon also applies to FbsA immunization.

Irrespectively of the mechanism, our data indicate that the 6p fragment of FbsA may be useful in anti-GBS immunization strategies, perhaps in conjunction with polysaccharidic or proteinaceous antigens, to increase efficacy and/or strain coverage. Indeed, the *fbsA* gene is widely present in GBS strains, having being detected in approximately 80% of human isolates [13] and in all strains belonging to the hypervirulent CC17 clone (which largely predominates in neonatal meningitis isolates [27]), or to the CC23 clone, which is also frequent in patients with invasive infections [15]. However, due to the presence of distinct regulatory systems in different strains (13), FbsA expression may vary considerably. Although immunization with 6p had a protective effect on the two GBS strains tested, future studies should be performed to assess the efficacy of FbsA/6p immunization against a wide variety of clinical isolates.

The ability to avidly bind Fng and fibrin is a feature of many Gram-positive extracellular pathogens and may represent a common, conserved mechanism to penetrate epithelial and endothelial barriers and/or escape phagocytic killing [21,28]. Streptococcal or staphylococcal mutants lacking Fng-binding proteins (or the Fng-binding regions of these proteins) are generally hampered in the ability to produce invasive disease [7,29]. Despite this, it has been sometimes difficult to prove that Fng-binding proteins promote virulence by actually binding Fng *in vivo*. For example, group A streptococcal mutants lacking the Fng-binding region of M5 protein are attenuated even in Fng-deficient mice, suggesting that this region might have functions other than Fng binding activity [30]. Similarly, although deletion of *fbsA* resulted in the attenuation of GBS virulence [11], it is presently unclear whether this effect is actually linked to the ability of FbsA to bind Fng *in vivo*. Such ability may indeed be crucial for GBS virulence, since administration of neutralizing anti-FbsA IgG or F(ab’)_2_ fragments significantly protected pups from lethal GBS challenge. In sharp contrast, a non-neutralizing anti-FbsA mAb was totally devoid of protective activity. These data are in general agreement with a previous study in which Fab fragments of FbsA-specific antibodies were as effective as the unfragmented IgG in preventing GBS-induced lethality in adult mice [18]. All together, we suggest that the protective activity of anti-FbsA antibodies is related to the interference with Fng binding, but not to opsonophagocytic killing, and that induction or administration of neutralizing anti-FbsA antibodies may be useful at preventing lethal sepsis by GBS. In conclusion, 6p FbsA fragment may be useful in the development of anti-GBS vaccines. Moreover, blocking Fng-FbsA interactions by passive immunization may be a viable strategy to prevent or treat GBS disease, particularly in the neonate where comparatively small doses of antibodies would be needed.

## Materials and Methods

### Bacterial strains and materials

GBS serotype III COH1 [31] and 6313 [32] strains and serotype V 2603V/R strain [33] were used in this study. A previously described 6313 mutant lacking FbsA (Δ*fbsA* mutant) [11] was also used in virulence studies. GBS were grown at 37°C in Todd-Hewitt broth containing 1% yeast extract. Human fibrinogen (Fng) was prepared as previously described [16].

### Construction and selection of *S. agalactiae* phage displayed libraries

For construction of genomic *S. agalactiae* λ phage displayed libraries, we used previously described procedures [25,34,35,36]. Briefly, 5 µg of streptococcal genomic DNA, obtained using standard phenol extraction procedures, were digested using 1 ng of DNaseI (Dnase shotgun cleavage kit, Novagen, Milan, Italy) at 16°C for 20 min. Fragments with an average size of approximately 300 bps were then manually cut from gels, filled in with T4 DNA polymerase (M4211; Promega, Milan, Italy) and ligated with specific adaptors into vector **λ**KM4, to be cloned as fusion products with the coat λ phage protein D [34]. The resulting COH1 and 2603 V/R displayed libraries contained, respectively, 4 x 10^6^ and 2 x 10^6^ independent recombinant phages, thus providing full coverage of the genomes of these strains. Selection with Fng was performed using Fng-coated magnetic beads (Dynabeads M-280, Invitrogen, Monza, Italy). Coating was performed by incubating 5 mg of tosyl-activated magnetic beads overnight at 37°C with 50 µg of Fng in 500 µl of borate buffer. Before use, the beads were blocked by incubation with 1 ml of PBS/milk (0.05 M phosphate buffered saline supplemented with 5% non-fat dry milk) for 3h at 20°C, and were reacted for 1h at 20°C with phages from the COH1 or 2603V/R libraries (10^10^ PFU in 1 ml). After washing, *Escherichia coli* LE392 cells, grown to the exponential phase, were infected with Fng-selected phages and plated onto LB agar. Plaque immunoscreening was performed as previously described [34] using Fng (5µg/ml) followed by rabbit anti-Fng IgG (diluted 1:64,000) (Abcam, Cambridge, UK) and alkaline phosphatase-conjugated goat anti-rabbit IgG (diluted 1:2,000, Sigma, Milan, Italy). After selection, libraries or individual phage clones were analyzed by a phage enzyme-linked immunoassay (phage ELISA [34]). Briefly, plates were sensitized with Fng (5 µg/ml) followed by the addition of a 100 µl suspension containing the indicated numbers of phage particles. Next, anti-lambda rabbit IgG was added followed by alkaline phosphatase-conjugated goat anti-rabbit IgG, as described [25,35]. Phage ELISA inhibition assays were performed by mixing phages (10^8^ PFU) with various concentrations of recombinant FbsA fragments (see below), used as inhibitors.

### Production of recombinant FbsA fragments

FbsA fragments expressed on the surface of selected phage clones were recombinantly produced as previously described [25,36]. Briefly, inserts from the Fng-binding clones 5p and 6p (see Results section) were amplified and subcloned into the pGEX-SN bacterial expression vector [37], to produce pGEX-SN5p and pGEX-SN6p that allowed the expression of recombinant proteins as fusions to glutathione S-transferase (GST). After induction, recombinant fragments were purified from the cytoplasm of bacterial cells using affinity chromatography [36]. Recombinant GST, to be used as a negative control, was produced and purified using the same method.

### Generation of monoclonal and polyclonal antibodies

Monoclonal antibodies 5H2 and 10H1, both IgG_1k_, were raised against an FbsA-derived synthetic peptide as previously described [16]. 5H2 F(ab’)_2_ fragments were prepared by digestion of whole 5H2 IgG with immobilized pepsin, according to the manufacturer’s instructions (Pierce, Rockford, IL, USA). Mouse polyclonal antiserum against human Fng was generated by injecting BALB/c mice intraperitoneally (i.p.) four times at 1-week intervals with 50 µg of the purified protein. The antigen was emulsified with an equal volume of complete Freund’s adjuvant for the first immunization, followed by three injections in incomplete adjuvant. The mice were bled, and the sera were tested for reactivity to the purified antigen using ELISA and Western blot. The use of complete Freund’s adjuvant in the first immunization was justified by our previous observations that high-titer sera were more consistently obtained with this adjuvant, as compared to other less “inflammatory” adjuvants such as alum. However, care was taken to minimize discomfort to the animals by injecting a low volume (0.1 ml containing 0.05 mg of mycobacteria) of the oily component of the emulsion and by using sterile solutions and techniques to prepare it. Under these conditions, no significant abdominal distension or other complications at the injection site were observed throughout the experimental period.

To produce rabbit anti-GBS antibodies, cells of *S. agalactiae* R268 were inactivated with 0.8% formol for 48 hours under stirring conditions. Bacteria were harvested, washed with phosphate-buffered saline and injected intramuscularly into a rabbit. The primary immunization consisted of 1 ml of bacteria (1.5x10^9^) emulsified with an equal volume of complete Freund adjuvant. Booster doses of streptococci mixed with incomplete adjuvant were administered by the same route 21, 35, 50 and 65 days after the primary dose. Serum obtained from bled animal was tested for reactivity to GBS by ELISA. Control serum was obtained prior to animal inoculation. The antibodies were purified by affinity chromatography on Protein A/G-Sepharose columns according to the recommendations of the manufacturer (GE Healthcare, Milan, Italy). Horseradish peroxidase (HRP)-conjugated rabbit anti-mouse antibody and horseradish peroxidase-conjugated goat anti-rabbit IgG were both from Dako (Glostrup, Denmark). Alkaline phosphatase-conjugated goat anti-rabbit IgG were purchased from Sigma.

### Analysis of Fng binding to recombinant fragments of FbsA by ELISA

Saturation kinetics of Fng-binding to recombinant FbsA-fragments were determined by an ELISA assay. Briefly, 5pGST or 6pGST (500 ng/well) were coated onto microtiter wells overnight at 4°C in 0.1 M carbonate buffer. The wells were washed, blocked with 2% BSA for 1 h at 22°C and incubated with increasing amounts of human Fng. Complex formation was detected with mouse anti-human Fng IgG (0.3 μg/well), followed by addition of peroxidase-conjugated rabbit anti-mouse IgG (1:1000).

### Surface plasmon resonance studies

Surface plasmon resonance (SPR) was determined using the BIAcore X system (GE Healthcare). To measure K_D_ values of Fng binding to recombinant FbsA fragments (5pGST and 6pGST), goat anti-GST antibody (30 µg/ml) dissolved in 10 mM sodium acetate buffer, pH 5.0 was immobilized onto a carboxy-derivatized sensor chip. The 5pGST or 6pGST (500 nM) were passed over a flow cell, while GST alone was passed in a reference cell. Human Fng was then flowed over the surface of both flow cells at concentrations ranging from 2.92 nM to 750 nM at a rate of 20 µl/min. Assay channel data was subtracted from reference flow cell data to eliminate the effects of non-specific interactions. The data were analyzed using the BIA evaluation software version 3.0. A plot of the level of binding (response units) at equilibrium against analyte concentration was used to determine K_D_ values.

### Inhibitory effect of monoclonal antibodies on GBS attachment to Fng

Microtiter plates were coated overnight at 4°C with 1 µg human purified Fng in 100 µl of PBS. The wells were washed three times with phosphate–buffered-saline and then blocked with 1% bovine serum albumin for 2 hours. Then, 5x10^7^ cells of GBS strain 6313, preincubated with the indicated amounts of anti-FbsA monoclonal antibodies, were added to each well and the mixtures incubated for 2 hours at 22°C. After extensive washing of the plates, 1 µg rabbit anti-*S. agalactiae* IgG was added to each well, followed by a further incubation for 90 minutes. Subsequently, peroxidase-conjugated goat anti-rabbit IgG was added, and a color reaction developed after the addition of o- phenylenediamine. Absorbance at 490 nm was quantified in a microplate reader (Bio-Rad, Segrate, Milan, Italy).

### Active immunization and challenge of adult mice

To study the protective activity of 6pGST immunization in an adult mouse sepsis model, CD1 mice (5 wk old), (Charles River Labs, Calco, Italy) were injected i.p. with 20 µg of 6pGST, or of GST used as a control, in complete (first injection) or incomplete (second and third injections) Freund’s adjuvant emulsions (in a total volume of 0.2 ml) on day 0, 14, and 28. Three weeks after the last immunization, mice were challenged i.p. with the COH1 GBS strain (5×10^7^ CFUs) and bled after 18h from the tail vein to measure blood CFUs by agar plating. Mice were monitored at least once a day for lethality and signs of disease for a total of 14 days after challenge. Animals with signs of irreversible sepsis were humanely euthanized and their organs were cultured to confirm GBS as the cause of disease.

### Maternal immunization

A previously described murine maternal immunization model was used [19]. Briefly, female CD1 mice (5 wk old) were injected i.p. with 20 µg of 6pGST, or GST alone, exactly as described above. Mice were then time-mated at 2 weeks after the last immunization. Two-day old pups were infected subcutaneously (s.c.) with 100 to 250 CFUs of strain 6313 (grown as described above) and observed for disease signs and lethality for at least 10 days. Deaths never occurred, however, after 4 days.

### Passive protection model

In passive protection experiments, neonatal mice from non-immunized mothers were injected s.c. with polyclonal mouse sera or with monoclonal antibodies before challenge. Polyclonal mouse sera were obtained by immunizing CD1 mice (5 wk old) with 6pGST or GST, as described above. After bleeding mice at 2 weeks after the last injection, sera were pooled, frozen into 50 µl aliquots and used at a 1:10 dilution in PBS for injection of neonatal mice (30 µl per pup). Purified monoclonal antibodies and (Fab’)_2_ fragments were adjusted to 1 and 0.66 mg/ml concentrations in PBS, respectively, before the s.c. administration of 30 µl per pup. At 3 h after antibody administration, pups were challenged with 100 or 250 CFUs of *S. agalactiae* strain 6313. Survival was observed for 10 days. Deaths never occurred, however, after 4 days.

### In vivo effects of fbsA deletion

To assess the role played by FbsA in GBS virulence, a Δ*fbsA* deletion mutant and the parental 6313 strain were used to infect adult or neonatal mice. Bacteria were grown to the mid log phase (OD_600_ = 0.6), washed and plated for colony counts. Eight-week old CD1 mice were inoculated i.p. with 2×10^8^ CFUs. Two-day-old pups were challenged with 250 CFUs administered by the s.c. route.

### Ethics statement

All *in vivo* experiments were conducted at the animal facilities of the Dipartimento di Scienze Pediatriche, Ginecologiche, Microbiologiche e Biomediche of the University of Messina according to the European Union guidelines for the handling of laboratory animals and were approved by the local animal experimentation committee (Comitato Etico per la Sperimentazione Animale, permit 18052010).

## Supporting Information

Figure S1 **Blood CFUs in mice immunized with the 6pFbsA fragment.** Blood samples were obtained at 18 h after challenge from the animals used in Figure 4A experiments. CFUs were counted by plating serial dilutions on blood agar. For the purpose of statistical analysis, samples in which no CFU were detected were assigned an arbitrary value corresponding to one half of the lower detection limit of the assay. *, p<0.05 relative to GST-immunized mice by one-way ANOVA and the Student-Keuks-Newman test. Shown are the cumulative results of two independent experiments.
(TIF)

Figure S2 **Inhibition of Fng binding to 6pGST by the 5H2 mAb.** Goat anti-GST was immobilized on the wells of microtiter plates, followed by incubation with 6pGST (100 nM). Fng (500 nM) was mixed with 1 µg/ml of mAb 5H2, mAb 10H1 or mouse IgG1 (isotype control) before being added to the wells. After washing, Fng binding was detected using rabbit anti-Fng antibodies followed by alkaline phosphatase-conjugated goat anti-rabbit IgG.
(TIF)

Figure S3 **Inhibition of GBS attachment to surface-coated Fng by the 5H2 mAb.** Cells of *S. agalactiae* 6313 (5x10^7^) were preincubated with the indicated amounts of mAbs 5H2 or 10H1, transferred to Fng-coated wells (1 µg/well) and the mixtures were incubated for 2 hours. After extensive washes, 1 µg rabbit anti-GBS IgG was added to the wells, followed by a 90 min incubation. Adherent bacteria were detected by peroxidase-conjugated goat anti-rabbit IgG and the plates were developed with o*-phenylenediamine*. All the data are expressed as percentages of control adherence, where bacteria attachment in the absence of antibody was set to 100% (equivalent to 0% inhibition). The bars show standard deviations of triplicate samples. This experiment was performed three times with similar results.
(TIF)

Figure S4 **Effects of passive immunization with 5H2 F(ab’)_2_ fragments in a neonatal mouse model of GBS sepsis.** Two-day-old pups born to unimmunized mothers were administered with equimolar amounts of full length IgG (5H2 IgG or isotype control IgG_1k_, 30 µg per animal) or with 5H2 F(ab’)_2_ fragments (20 µg per animal) via s.c. route. After 3 h, pups were infected s.c. with 250 CFUs of GBS strain 6313. *, p<0.05 relative to control IgG treated-mice by Kaplan-Meier survival plots.
(TIF)

Table S1 **Amino acid sequences of inserts of FbsA phage clones.** DNA was amplified from the indicated phage clones (left column) and sequenced. Deduced amino acid sequences are listed in the right column.
(TIF)
