# Supplementary figures and images for: Immunogenic Properties of Streptococcus agalactiae FbsA Fragments
Source: PLoS One. 2013 Sep 24;8(9):e75266. doi: 10.1371/journal.pone.0075266 (PMC3782484; doi:10.1371/journal.pone.0075266)

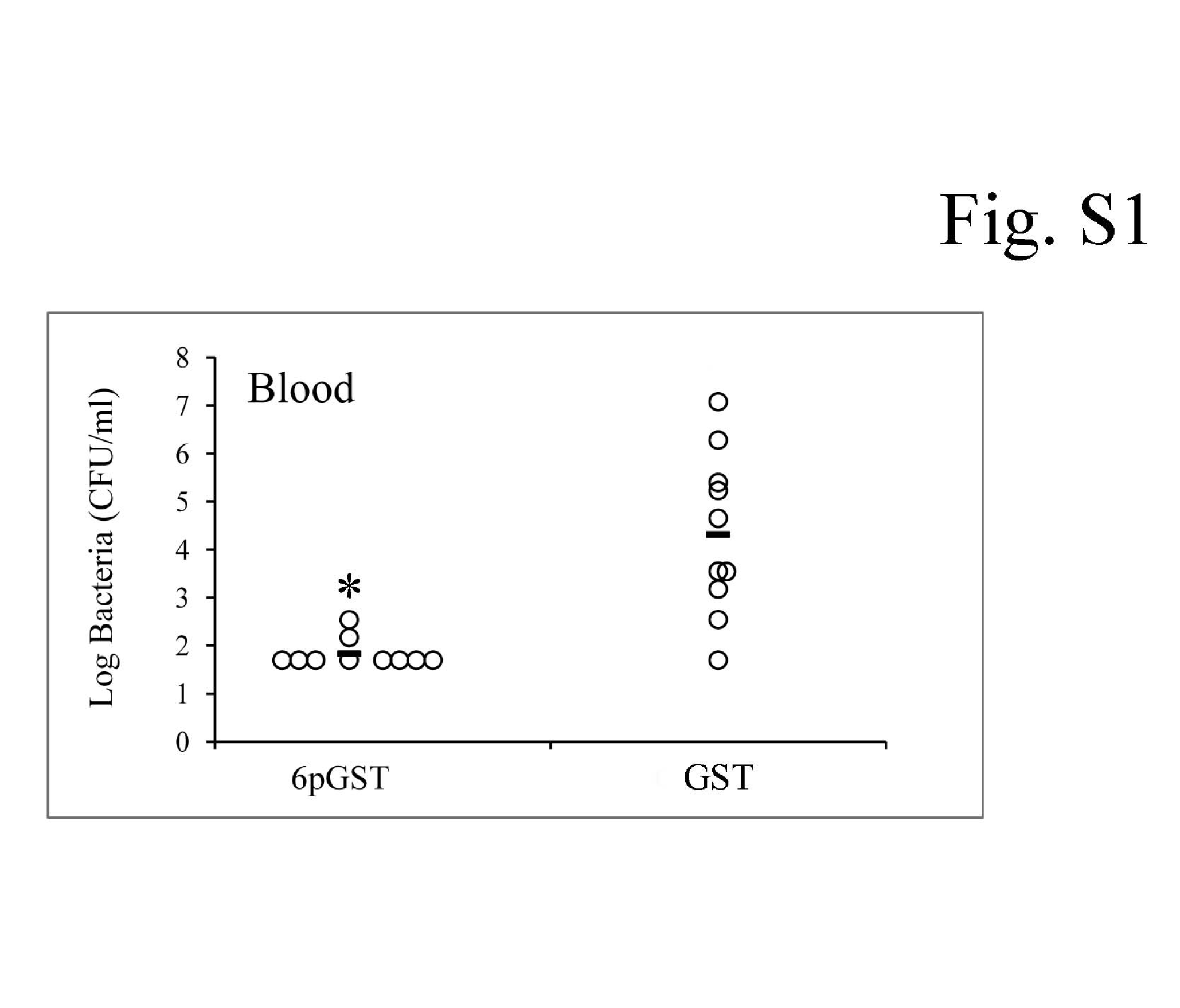

Supplement: Figure S1 — Blood CFUs in mice immunized with the 6pFbsA fragment. Blood samples were obtained at 18 h after challenge from the animals used in Figure 4A experiments. CFUs were counted by plating serial dilutions on blood agar. For the purpose of statistical analysis, samples in which no CFU were detected were assigned an arbitrary value corresponding to one half of the lower detection limit of the assay. *, p<0.05 relative to GST-immunized mice by one-way ANOVA and the Student-Keuks-Newman test. Shown are the cumulative results of two independent experiments. (TIF) [file pone.0075266.s001.tif]

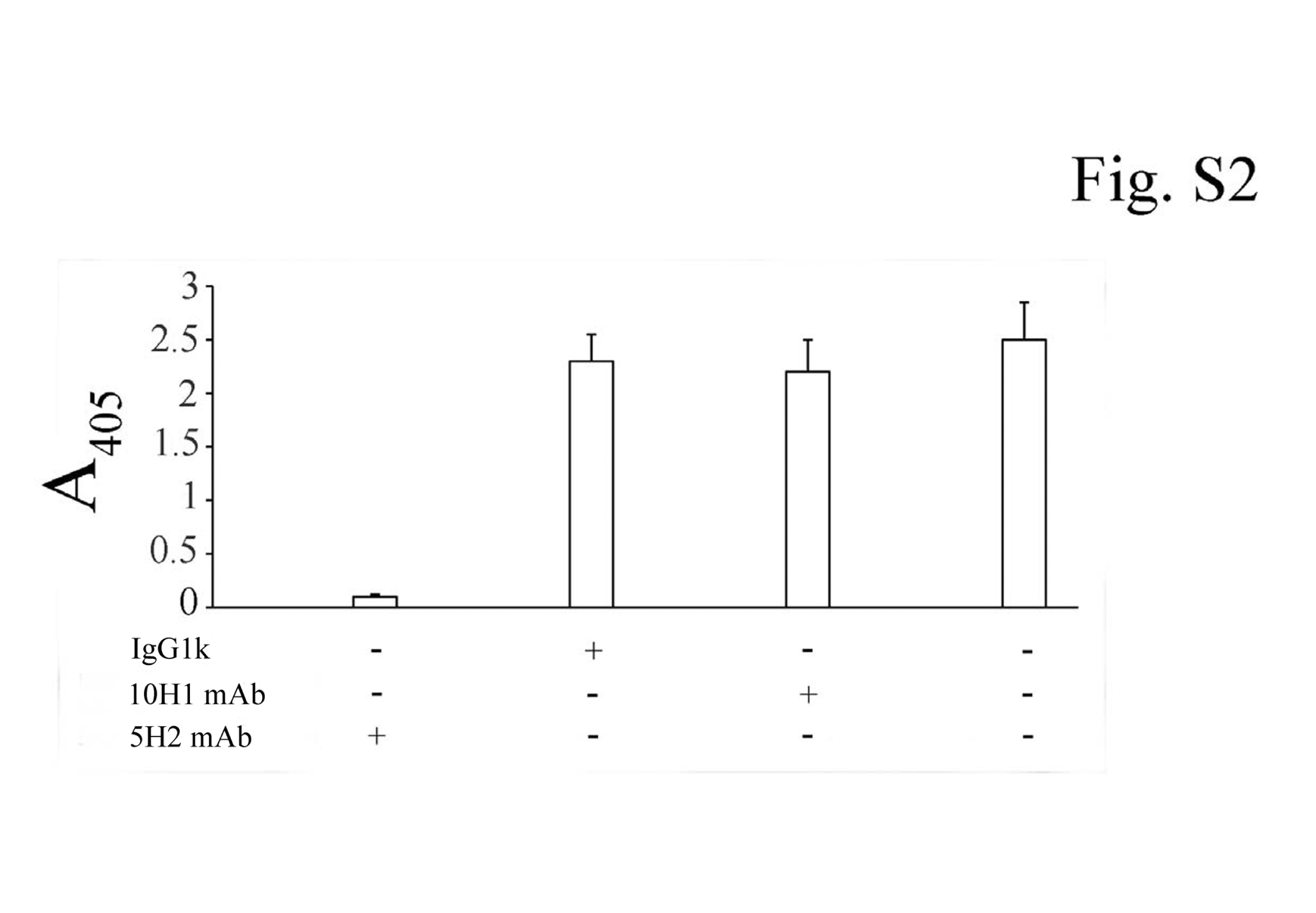

Supplement: Figure S2 — Inhibition of Fng binding to 6pGST by the 5H2 mAb. Goat anti-GST was immobilized on the wells of microtiter plates, followed by incubation with 6pGST (100 nM). Fng (500 nM) was mixed with 1 µg/ml of mAb 5H2, mAb 10H1 or mouse IgG1 (isotype control) before being added to the wells. After washing, Fng binding was detected using rabbit anti-Fng antibodies followed by alkaline phosphatase-conjugated goat anti-rabbit IgG. (TIF) [file pone.0075266.s002.tif]

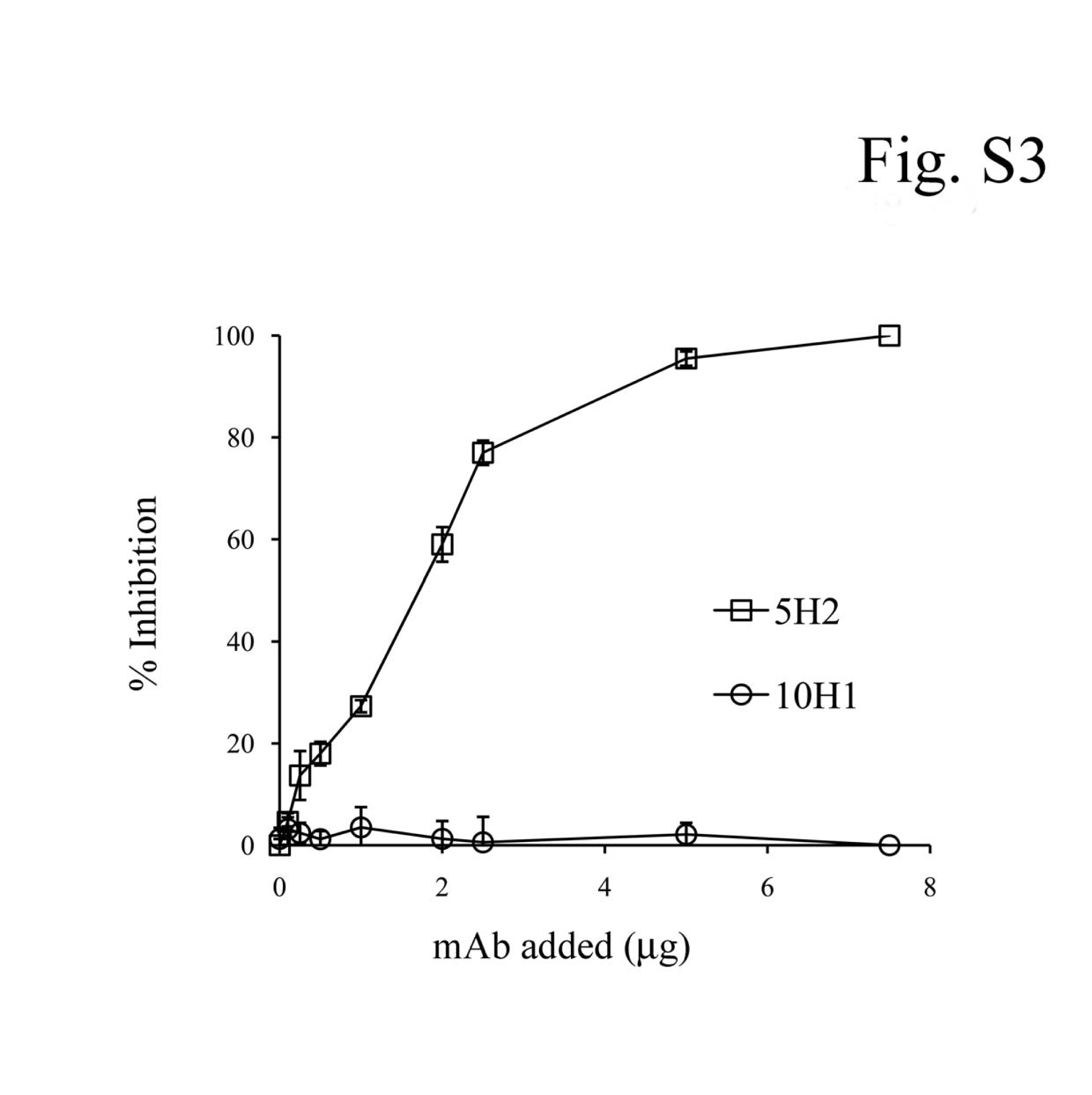

Supplement: Figure S3 — Inhibition of GBS attachment to surface-coated Fng by the 5H2 mAb. Cells of S. agalactiae 6313 (5x10^7^) were preincubated with the indicated amounts of mAbs 5H2 or 10H1, transferred to Fng-coated wells (1 µg/well) and the mixtures were incubated for 2 hours. After extensive washes, 1 µg rabbit anti-GBS IgG was added to the wells, followed by a 90 min incubation. Adherent bacteria were detected by peroxidase-conjugated goat anti-rabbit IgG and the plates were developed with o-phenylenediamine. All the data are expressed as percentages of control adherence, where bacteria attachment in the absence of antibody was set to 100% (equivalent to 0% inhibition). The bars show standard deviations of triplicate samples. This experiment was performed three times with similar results. (TIF) [file pone.0075266.s003.tif]

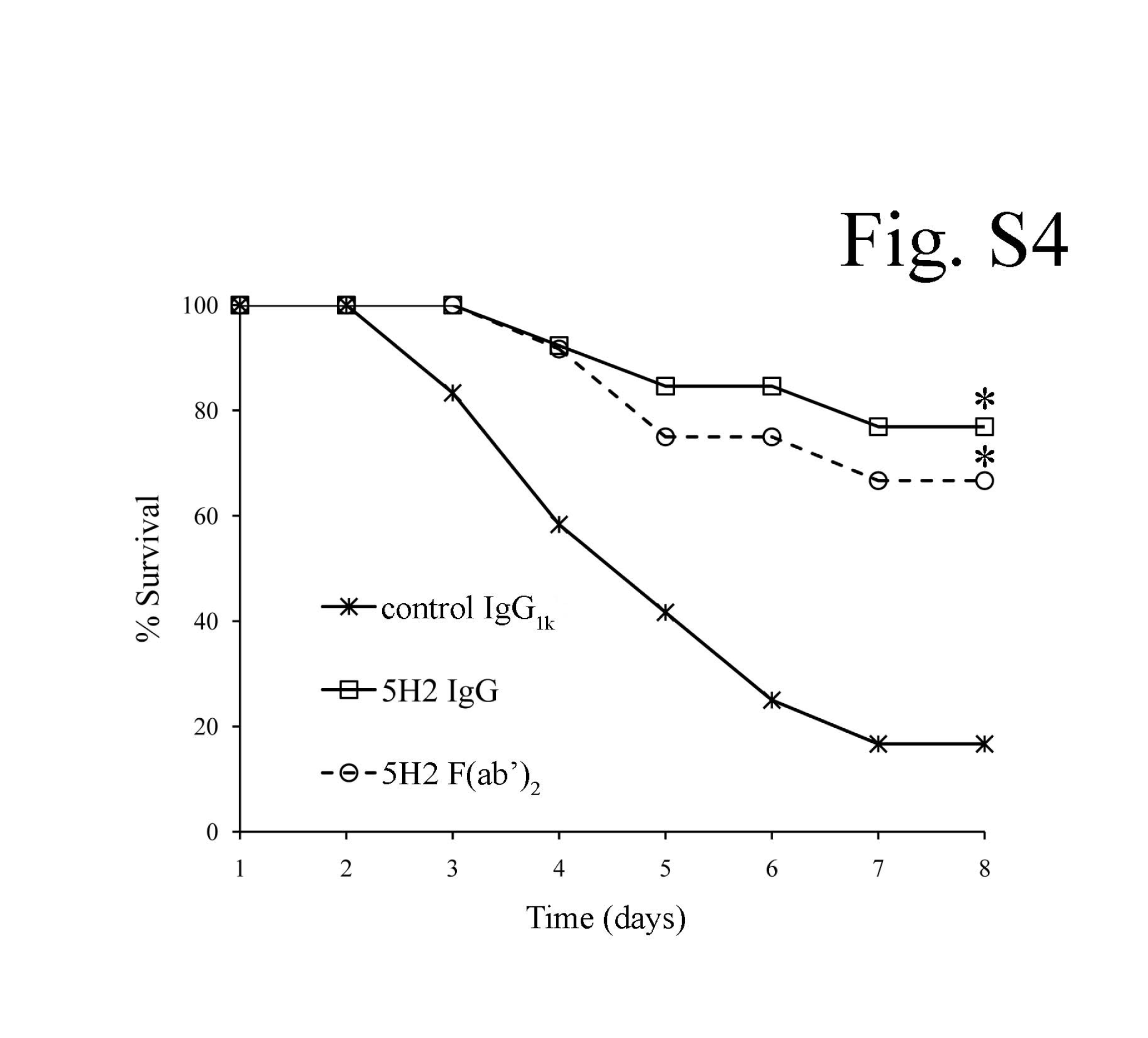

Supplement: Figure S4 — Effects of passive immunization with 5H2 F(ab’)2 fragments in a neonatal mouse model of GBS sepsis. Two-day-old pups born to unimmunized mothers were administered with equimolar amounts of full length IgG (5H2 IgG or isotype control IgG1k, 30 µg per animal) or with 5H2 F(ab’)2 fragments (20 µg per animal) via s.c. route. After 3 h, pups were infected s.c. with 250 CFUs of GBS strain 6313. *, p<0.05 relative to control IgG treated-mice by Kaplan-Meier survival plots. (TIF) [file pone.0075266.s004.tif]

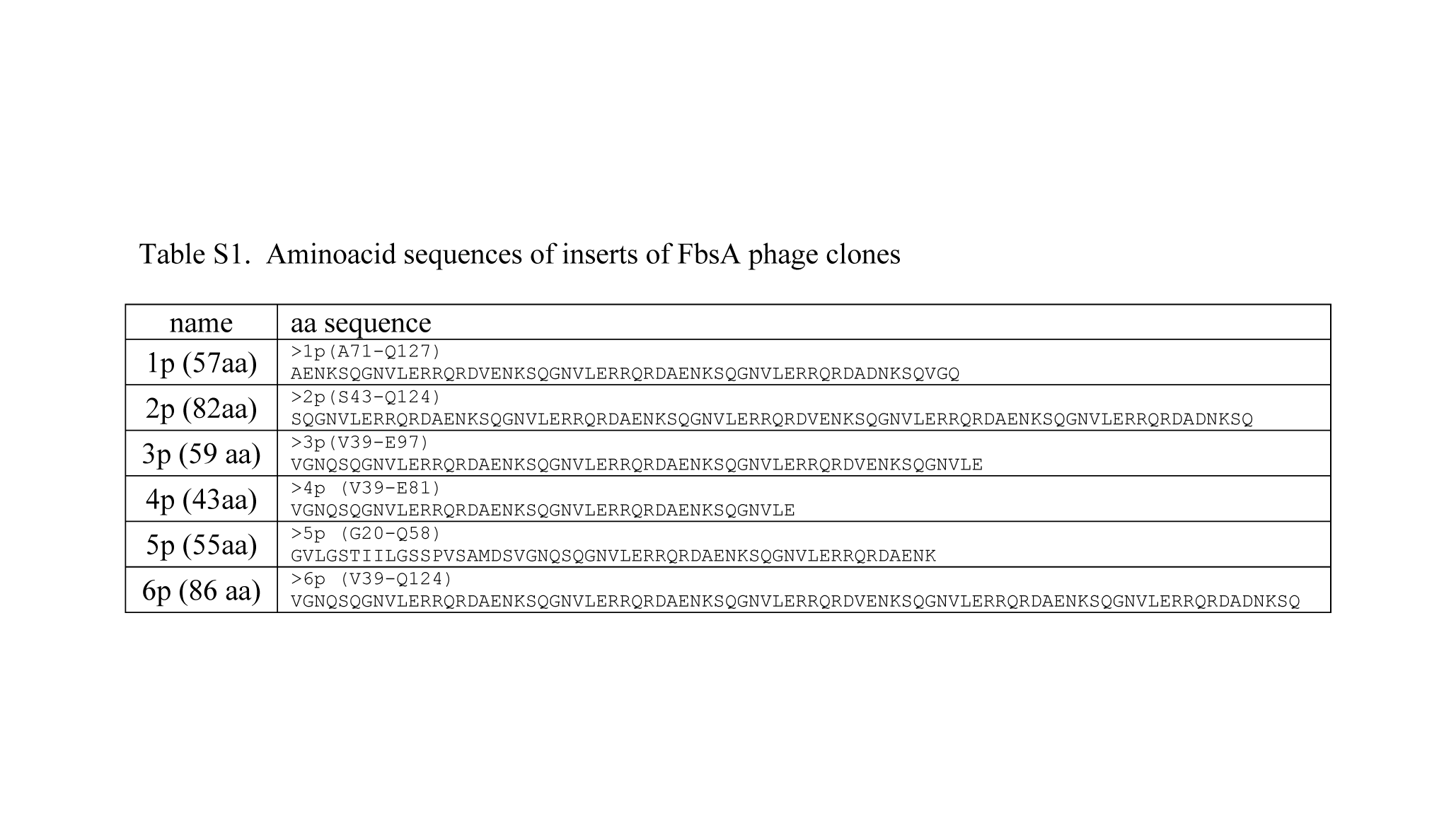

Supplement: Table S1 — Amino acid sequences of inserts of FbsA phage clones. DNA was amplified from the indicated phage clones (left column) and sequenced. Deduced amino acid sequences are listed in the right column. (TIF) [file pone.0075266.s005.tif]
